# Real world genomic profiling in non-small cell lung cancer reveals regional variations with clinical implications

**DOI:** 10.3389/fonc.2026.1882423

**Published:** 2026-09-22

**Authors:** Nicholas Karlowatz, Linda Köhn, Cedric Patthey, Jonas Nilsson, Mikael B Johansson

**Affiliations:** 1 Department of Diagnostics and Intervention, Oncology, Umeå University, Umeå, Sweden; 2 Department of Medical Biosciences, Umeå University, Umeå, Sweden

**Keywords:** clinical genomics, lung cancer, mutational landscape, North Sweden, precision oncology, somatic mutation, targeted profiling

## Abstract

**Background:**

Routine targeted genomic profiling in non-small cell lung cancer (NSCLC) is primarily used to identify actionable alterations but may provide more information for clinical decision making. In this study routine genomic profiling data were used to examine the mutational landscape of lung cancer and its relationship to patient characteristics.

**Materials and methods:**

We analyzed Archer VariantPlex 67-gene sequencing data from 528 patients diagnosed with lung cancer in the North Swedish Health Care Region between 2011 and 2021 and linked the genomic results to registry-derived clinical and demographic data. Mutation frequencies, positional architectures, subgroup enrichments, and survival associations were compared within the regional cohort and against an external reference cohort assembled from cBioPortal.

**Results:**

After quality filtering, 1,415 somatic variants across 60 genes were identified, with a mean of 2.68 mutations per tumor, indicating substantial heterogeneity in mutational profiles. TP53 and KRAS formed a conserved mutational backbone across cohorts, whereas EGFR was less frequent and MET, APC, ATM, and CDKN2A were enriched in North Sweden relative to the external reference cohort. Major hotspot architectures were broadly preserved, although MET showed the clearest positional divergence. Mutation patterns varied by age, sex, smoking status, and disease stage. EGFR defined the most coherent clinical subtype, being enriched in females, never-smokers, younger patients, and earlier-stage disease, whereas KRAS, STK11, and TP53 were associated with smoking. EGFR was the strongest favorable prognostic marker, but stage strongly influenced the interpretation of many survival differences, indicating that gene-level prognostic signals are closely shaped by clinical context.

**Conclusions:**

Routine genomic profiling in NSCLC provides clinically relevant information beyond actionable driver detection when interpreted together with stage, smoking history, age, sex, and regional molecular context. These findings support a more context-aware interpretation of routine molecular diagnostics in thoracic oncology.

## Graphical Abstract

**Figure:**
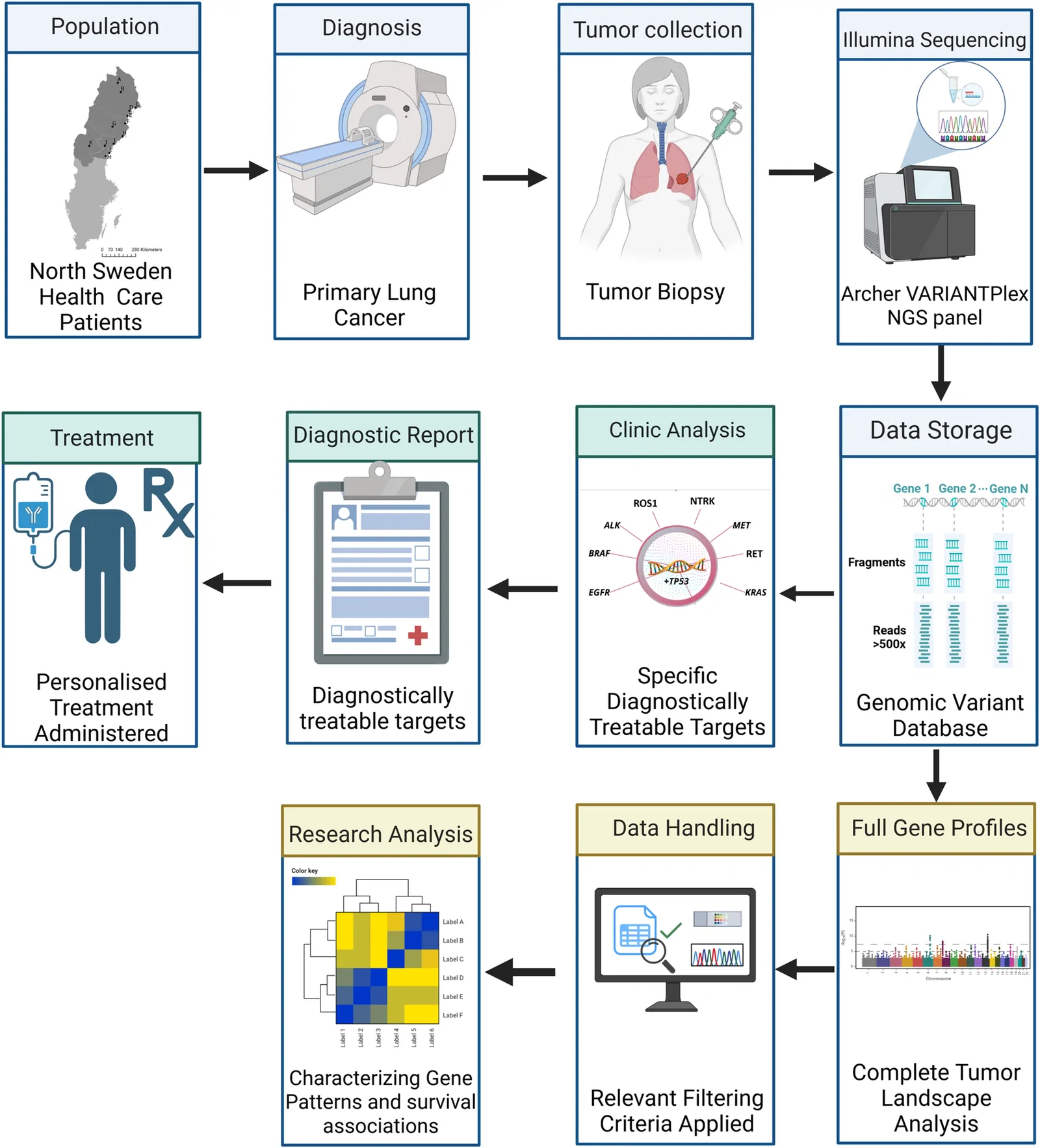
Workflow of the study. North Swedish Health Care Region patients with primary lung cancer underwent routine targeted sequencing with the Archer VariantPlex 67-gene panel (Illumina NextSeq 550) and sequencing data was processed using the Archer^®^ Analysis software. After clinicopathological and variant-level filtering, the final analytic cohort comprised 528 patients with linked survival data from the National Lung Cancer Registry, enabling cohort-level mutation profiling, comparison with a harmonised external reference cohort from cBioPortal, and exploratory survival analyses stratified by gene mutation status and clinical subgroup.

## Introduction

Lung cancer remains one of the leading causes of cancer-related mortality in Sweden, with approximately 4,500 new cases resulting in 3,500 deaths annually (1, 2). Its development is influenced by a complex interplay of environmental, genetic, and demographic factors (1, 3). Molecular profiling is crucial for lung cancer management, enabling personalised treatment strategies based on specific targetable alterations identified at diagnosis (2).

Smoking still remains the dominant environmental risk factor and approximately 85% of all lung cancer cases arise in current or former smokers (1, 3–5). However, its relative contribution is shifting as non-smoking-related adenocarcinoma rises globally (1, 3–5). Other risk factors for lung cancer are becoming more important and genetic predisposition as well as environmental factors will influence the landscape of lung cancer in the near future. Age and sex are critical factors influencing cancer risk and prognosis in general. As individuals age, the accumulation of molecular and cellular damage, such as genomic instability and chronic inflammation, creates a pro-tumorigenic environment (5). These aging-associated processes not only accelerate cancer initiation but also shape the mutational landscape, with distinct age-related mutation patterns observed in lung cancer. In *EGFR* mutated lung cancer, younger patients often present with *EGFR* exon 19 deletions, while L858R mutations are more prevalent in older patients (6). Additionally, sex-based differences, driven by genetic and hormonal factors, significantly impact cancer development. Females exhibit a protective effect against oncogenic mutations due to gene dosage effects, and higher levels of sex hormone receptor expression (particularly oestrogen receptors) in women are linked to specific molecular profiles, such as *EGFR* mutations (7, 8). Hormones like oestrogens and androgens modulate critical pathways involved in DNA repair and immune responses, influencing treatment strategies and outcomes (9, 10). Regional differences that encompass environmental exposures and lifestyle factors play a critical role in shaping the mutational landscape of lung cancer (11). Air pollution is an important risk factor for lung cancer and is reported to be of importance for the development of lung adenocarcinoma (12, 13).

This study characterises the somatic mutational landscape of lung cancer in North Sweden using routine genomic profiling data, compares the regional mutation profile to an international reference cohort, and correlates the prognostic information to stage, smoking history, age, and sex.

## Materials and methods

### Patient cohort and clinical data collection

All patients in the North Swedish Healthcare Region with primary lung cancer diagnosed between 2011 and 2021 who were examined on the regional molecular pathology platform using the 67-gene Archer VariantPlex^®^ Solid Tumour panel were included in the study. Profiling was performed as institutional reflex testing applied to all NSCLC patients regardless of histological subtype, and no selective profiling by histotype was carried out. Patients diagnosed between 2010 and 2015 were profiled retrospectively from archived FFPE material during platform implementation, while those diagnosed from 2015 onwards were profiled prospectively. Clinical and demographic data, including age, sex, smoking status, histological subtype, and disease stage, were obtained from the National Lung Cancer Registry (NLCR). Smoking status was classified as current, former, or never smokers. The study was approved by the Swedish Ethical Review Authority (2021-06549-01).

### Tissue acquisition and DNA extraction

Tumour tissue was obtained via standard clinical biopsy procedures, including fine needle aspiration, core needle biopsy, bronchoscopic biopsy and surgical resection specimens. Cytological slides and cell blocks were also included where tumour cellularity requirements were met. Samples were processed as formalin-fixed paraffin-embedded (FFPE) tissue blocks or cytological slides. Tumour content was assessed by a pathologist, and only samples with tumour cellularity > 10% were included in the final study cohort. DNA was extracted using Formapure XL DNA according to the manufacturer’s instructions. DNA concentration and purity were measured using spectrophotometry and fluorometric quantification.

### Targeted sequencing and library preparation

Genomic profiling was performed using the Archer VariantPlex 67 gene panel (ArcherDX, Boulder, CO), which targets exonic, selected intronic, and promoter regions of 67 genes implicated in solid tumour oncology, including lung, colorectal, and other common malignancies. This is a multi-cancer panel used routinely in clinical molecular profiling of solid tumours at the department of Clinical Genetics, University Hospital of Umeå. This panel detects single-nucleotide variants and small insertions/deletions. Gene fusions are assessed separately by FISH/IHC per routine clinical protocol, and the panel does not capture CDKN2A Panel composition was consistent throughout the study period (2011–2021), though minor bioinformatics pipeline updates occurred over this interval and temporal batch effects cannot be fully excluded. Library preparation was conducted according to the manufacturer’s protocol. Briefly, 50–400 ng of genomic DNA was fragmented, end-repaired, and ligated to molecular barcoded adapters. Target enrichment was achieved by hybridization with biotinylated probes, followed by PCR amplification. Libraries were quantified, pooled, and sequenced on an Illumina NextSeq 550 platform using paired-end 150 bp reads.

### Bioinformatic processing and variant calling

Sequencing data were processed with Archer Analysis v.6.2.7 using the DNA SNP/InDel workflow. Processing was done with default settings except that Read Depth Normalization was set to 5M reads.

### Variant filtration and annotation

Variants were filtered to retain only high-confidence, functionally relevant alterations. Variants were excluded if the allele frequency (AF) exceeded 1% in the gnomAD database v3.1.2. Additional filters included removal of variants with AF <0.05, sequencing depth <100×, or fewer than five alternate reads. Known technical artefacts and variants with AF 100% were also excluded. Remaining variants were classified as missense, nonsense, frameshift, splice region, inframe InDel, or protein-altering, according to standard annotation guidelines.

### CBIO world cohort

External reference cohort patient data were systematically retrieved from cBioPortal by selecting tumour tissue samples from lung cancer cohorts with comprehensive mutation profiling and a minimum of 30 cases per study. Only mutation calls were included, and analyses were limited to studies of primary lung malignancies with attached survival data. The final cohort comprised five published datasets (14–18) downloaded 17-09-2024. These studies collectively represent both North American and Asian populations and provide whole-genome coverage for all genes in the targeted panel, ensuring robust and unified downstream survival and co-mutation analyses. As these five datasets span multiple ancestries, testing eras, and sequencing platforms, the reference cohort is used here as a broad contextual benchmark rather than a matched comparator, and gene-level differences should be interpreted with this heterogeneity in mind. Consistent with the Swedish cohort, the reference datasets also include non-adenocarcinoma histologies, limiting histology-driven comparison bias.

### Statistical analysis

Comparisons of mutation frequencies between the North Sweden cohort and external reference datasets were performed using chi-squared tests. Enrichment analyses were conducted to calculate fold changes in mutation frequencies across clinical and demographic subgroups, including sex, age, disease stage, and smoking status. Log2 fold change values were visualized in heatmaps, with colour scales indicating the direction and magnitude of enrichment. Kaplan-Meier survival analysis was performed using Python 3.12 and lifelines (v0.28.0). Survival curves were compared using the log-rank test, and hazard ratios with 95% confidence intervals were estimated using Cox proportional hazards regression. Formal multivariable survival modelling was not performed given limited subgroup sizes; clinical variables (sex, age, stage and smoking status) were instead examined through the enrichment analyses described above.

Volcano plots were generated to display the association between gene mutation status and 24-month survival probability, with the x-axis representing the difference in survival probability and the y-axis representing the –log10(p-value) from the log-rank test. For each gene, patients were stratified into mutated and non-mutated groups. Only genes mutated in at least five patients were included in survival analyses.

For positional architecture analyses of recurrent TP53, EGFR, KRAS, and MET alterations, two-sample Kolmogorov-Smirnov tests were used to compare the distributions of mutated amino-acid positions between the North Sweden and world cohorts. This was used to assess overall distributional similarity between cohorts in addition to hotspot-specific enrichment analyses.

Twenty-four-month survival probability was estimated by the Kaplan–Meier method for each gene within Stage 1–2 and Stage 3–4 groups. Somatic mutation prevalence was compared across sex, stage, smoking status, and age. Between-group differences were summarized as smoothed log2 fold changes using a Haldane–Anscombe-type pseudocount log2FC = log2(((a+0.5)/(n_red+1))/((c+0.5)/(n_blue+1) where a and c are the numbers of mutated patients in the red and blue groups and n_red and n_blue are the corresponding subgroup sizes. For inference, each gene-by-comparison was tested using a two-sided Fisher’s exact test on a 2×2 contingency table of mutation status by subgroup. Genes were retained for visualization if they had |log2FC|>0.5 in any comparison and/or p < 0.05 in any comparison.

## Results

A total of 603 patients from the North Swedish Health Care Region diagnosed with non–small cell lung cancer were matched to patients entered in the National Lung Cancer Registry (NLCR). Seventy–five patients were excluded due to non–primary lung cancer or failing quality criteria, resulting in a final study cohort of 528 patients (**Figure 1A**). The mean age at diagnosis was 71.26 years (median 72 years), and the cohort comprised 298 (56.4%) females and 230 (43.6%) males (**Table 1**). Smoking status varied, with 37.1% of patients classified as current smokers (196/528), 48.5% as former smokers (256/528), and 14.4% as never smokers (76/528).

**Figure 1:**
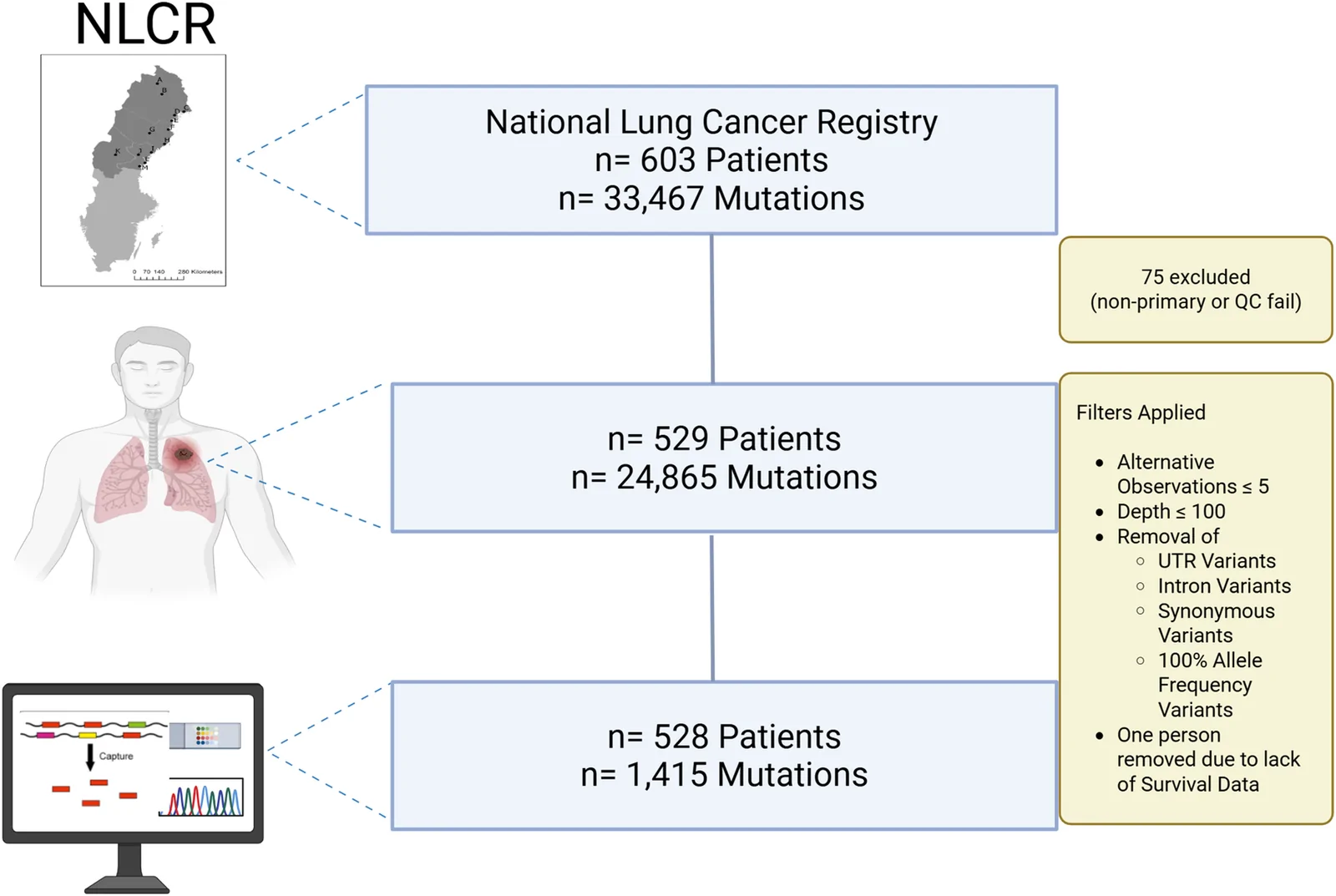
Patient selection and variant-filtering workflow for the North Swedish cohort. Registry-linked lung cancer cases were filtered for primary lung cancer diagnosis, sequencing quality, and analytic eligibility. The final survival-linked cohort comprised 528 patients with 1,415 retained somatic variants across 60 genes.

**Table 1:** Baseline characteristics of the North Sweden lung cancer cohort (N = 528).

Characteristic	Total (N=528)	Male (n=230)	Female (n=298)
Demographics			
Age, mean (median), years	71.26 (72)	71.51 (73)	71.07 (72)
Age below mean (71.26)	246 (46.6%)	99 (43.0%)	147 (49.3%)
Age above mean (71.26)	282 (53.4%)	131 (57.0%)	151 (50.7%)
Smoking Status			
Current smoker	196 (37.1%)	87 (37.8%)	109 (36.6%)
Former smoker	256 (48.5%)	118 (51.3%)	138 (46.3%)
Never smoker	76 (14.4%)	25 (10.9%)	51 (17.1%)
Disease Stage			
Stage I	139 (26.3%)	51 (22.2%)	88 (29.5%)
Stage II	44 (8.3%)	18 (7.8%)	26 (8.7%)
Stage III	101 (19.1%)	48 (20.9%)	53 (17.8%)
Stage IV	244 (46.2%)	113 (49.1%)	131 (44.0%)
Histological Subtype			
Adenocarcinoma	399 (75.6%)	159 (69.1%)	240 (80.5%)
Squamous cell carcinoma	100 (18.9%)	55 (23.9%)	45 (15.1%)
Large cell/Low-diff NSCLC	11 (2.1%)	7 (3.0%)	4 (1.3%)
Other/Unclassified	18 (3.4%)	9 (3.9%)	9 (3.0%)
Vital Status			
Alive	164 (31.1%)	61 (26.5%)	103 (34.6%)
Dead	362 (68.6%)	167 (72.6%)	195 (65.4%)
Unknown	2 (0.4%)	2 (0.9%)	0 (0.0%)
Median overall survival, months	23.3	19.8	31.0
Survival time distribution			
0-5 months	110 (20.8%)	53 (23.0%)	57 (19.1%)
6-20 months	130 (24.6%)	66 (28.7%)	64 (21.5%)
21-101 months	122 (23.1%)	48 (20.9%)	74 (24.8%)
Alive/unknown at last follow-up	166 (31.4%)	63 (27.4%)	103 (34.6%)

Shown are distributions of age, smoking status, disease stage, histological subtype, and survival, stratified by sex.

Most patients were diagnosed with adenocarcinoma (75.6%, 399/528), followed by squamous cell carcinoma (18.9%, 100/528) and other NSCLC subtypes (2.1%, 11/528) (**Table 1**). Stage distribution in the North Swedish cohort was dominated by advanced disease, with 244 patients (46.2%) presenting with stage IV and 101 patients (19.1%) with stage III disease, while smaller fractions were diagnosed at stage I (26.3%, 139/528) or stage II (8.3%, 44/528). Median overall survival was 23.3 months overall (19.8 months in males and 31.0 months in females). Survival time was distributed as follows: 20.8% of patients survived 0–5 months, 24.6% survived 6–20 months, 23.1% survived beyond 21 months, and 31.4% remained alive at last follow-up.

After filtering out normal variants, non-coding and synonymous sequence variants, and technical artifacts, 1,415 somatic sequence variants were included in the study, corresponding to 914 unique mutations across 60 genes, underscoring the substantial genomic heterogeneity (**Figure 1A**). Patients exhibited a mean of 2.68 mutations per person, with a range of 0–44 mutations and a median variant allele frequency of 0.237 (**Supplementary Table 1A**).

The global reference cohort, compiled from five cBioPortal lung cancer studies (14–18) (**Supplementary Figure 1B**), comprised 2,239 patients of whom 2,197 had survival data, and demonstrated a female predominance (54.1%) compared to males (45.9%) (**Supplementary Table 1A**). The full somatic mutational landscape of the world cohort is shown in **Supplementary Figure 1E**. The mean age within this cohort was 65.7 years however, age information was missing for 41.6% of patients. Early-stage disease was more common, with 26.8% diagnosed at stage I, whereas stage IV was rare (3.1% of males and 1.5% of females). This stage distribution is reflected in the comparatively favourable survival outcomes, as 69.1% of males and 75.8% of females were alive at the time of data collection. In contrast, the North Swedish cohort exhibited a higher proportion of patients presenting with advanced-stage disease and a correspondingly lower median survival of approximately 23 months.

### The North Swedish genetic landscape compared to a global reference cohort

Comparative analysis of mutation frequencies in North Sweden (n=528) and global (n=2,239) lung cancer cohorts revealed both shared and distinct patterns among the top mutated genes (**Figures 2A, B**). *TP53* was the most common mutation in both cohorts (56.6% vs 53.6%), with *KRAS* also frequent (28.8% vs 25.8%) (**Figures 2A, B**). *EGFR* mutations were significantly less common in North Sweden (13.6% vs 25.2, p < 0.001), while *CDKN2A* (9.5% vs 6.6%, p < 0.05), *APC* (13.3% vs 4.6%, p < 0.001), *ATM* (10.8% vs 6.6%, p < 0.01), and *MET* (8.3% vs 4.0%, p < 0.001) were enriched in the Swedish cohort. *STK11* (10.6% vs 11.6%) and *PIK3CA* (7.2% vs 7.4%) frequencies were similar. *BRAF* (4.2%), *ERBB4* (2.1%), and *KDR* (2.8%) were present in North Sweden at lower frequencies but appeared in both cohorts (**Figures 2A, B**).

**Figure 2:**
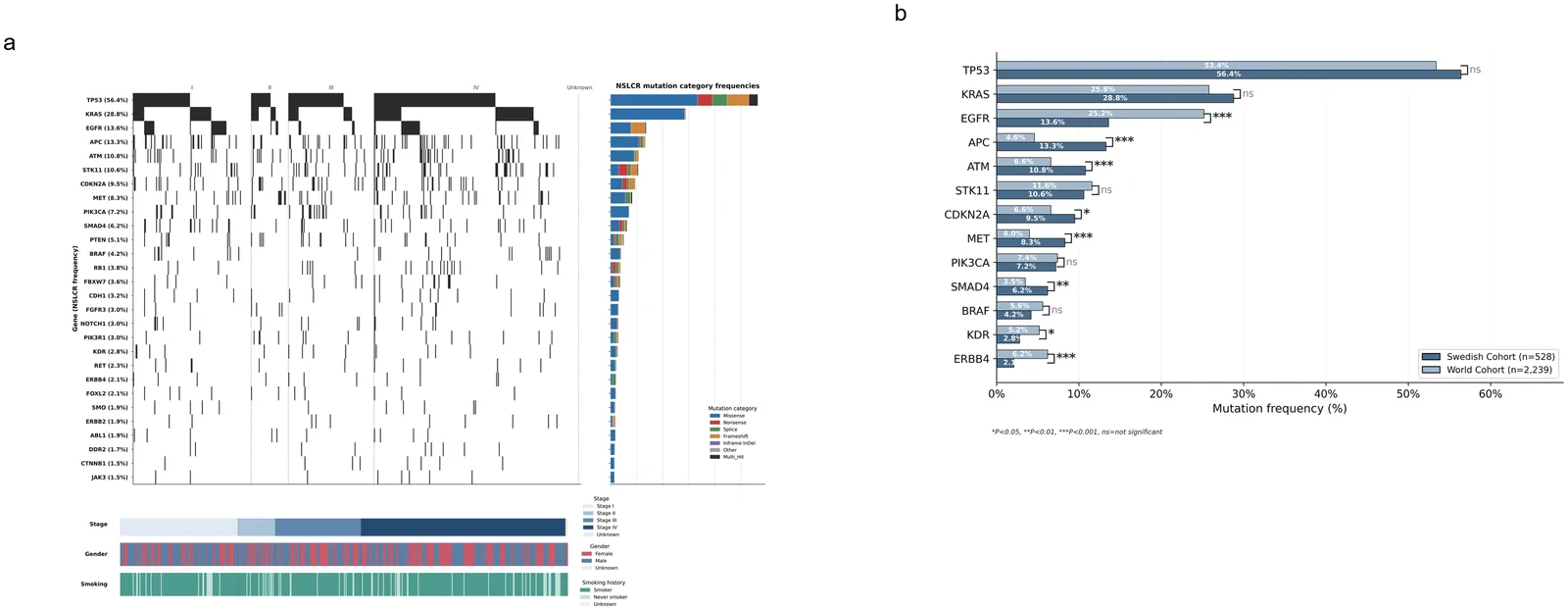
Somatic mutation landscape in the North Swedish and world lung cancer cohorts. **(A)** Heatmap showing recurrent gene alterations across the North Swedish cohort, with genes ordered by decreasing patient-level mutation frequency. Clinical annotation tracks for disease stage, sex, and smoking status are displayed beneath the mutation matrix. **(B)** Patient-level mutation frequencies for recurrent genes in the North Swedish cohort (dark blue; n=528) and the harmonised world cohort (light blue; n=2,239). Bars show the proportion of patients with at least one alteration in each gene. Significance markers indicate between-cohort frequency differences (*P<0.05, **P<0.01, ***P<0.001; ns, not significant).

Within North Sweden, stage-stratified clustering revealed a dominant *TP53*/*KRAS*-enriched subgroup across all stages, with *TP53* mutation frequency rising from 52.8% (stage I) to 71.4% (stage III) and 64.7% (stage IV), while *KRAS* showed no clear stage trend (29.9%, 35.7%, 24.2%, and 34.8% in stages I–IV respectively). Mutational burden remained steady across stages I–III (2.53, 2.61, 2.42) and only modestly higher in stage IV (2.69 mutations per patient, **Supplementary Figure 1A**). Rare events such as *EZH2, VHL*, and *RHOA* appeared predominantly in advanced stages.

Mutation-type distributions were broadly similar between cohorts with *TP53*, *STK11*, *CDKN2A*, and *ATM* showing nearly identical proportions of missense, frameshift, nonsense, and splice variants (all p > 0.05). *EGFR* and *BRAF* were dominated by missense and inframe InDels, and *KRAS* mutations were almost exclusively missense in both groups (**Figure 2A**).

*TP53* mutations were similarly prevalent in Sweden and globally (56.63% vs 53.60% of patients), and the positional landscape was highly conserved, with mutations clustering in the DNA-binding domain (**Figure 3A**). A two-sample Kolmogorov–Smirnov (KS) comparison of mutated codon positions supported similar overall positional and diversity profiles between cohorts (**Figure 3A**). Within this shared pattern, North Sweden showed localized enrichment at several codons, most notably R154X (4.35% of *TP53*-mutated Swedish cases vs 1.51% globally; odds ratio (OR) 2.97, p = 0.0049) and Y220C (codon 220; 3.01% vs 1.17%; OR 2.62, p = 0.032), while canonical global hotspots such as *R273* were comparable or slightly lower in Sweden.

**Figure 3:**
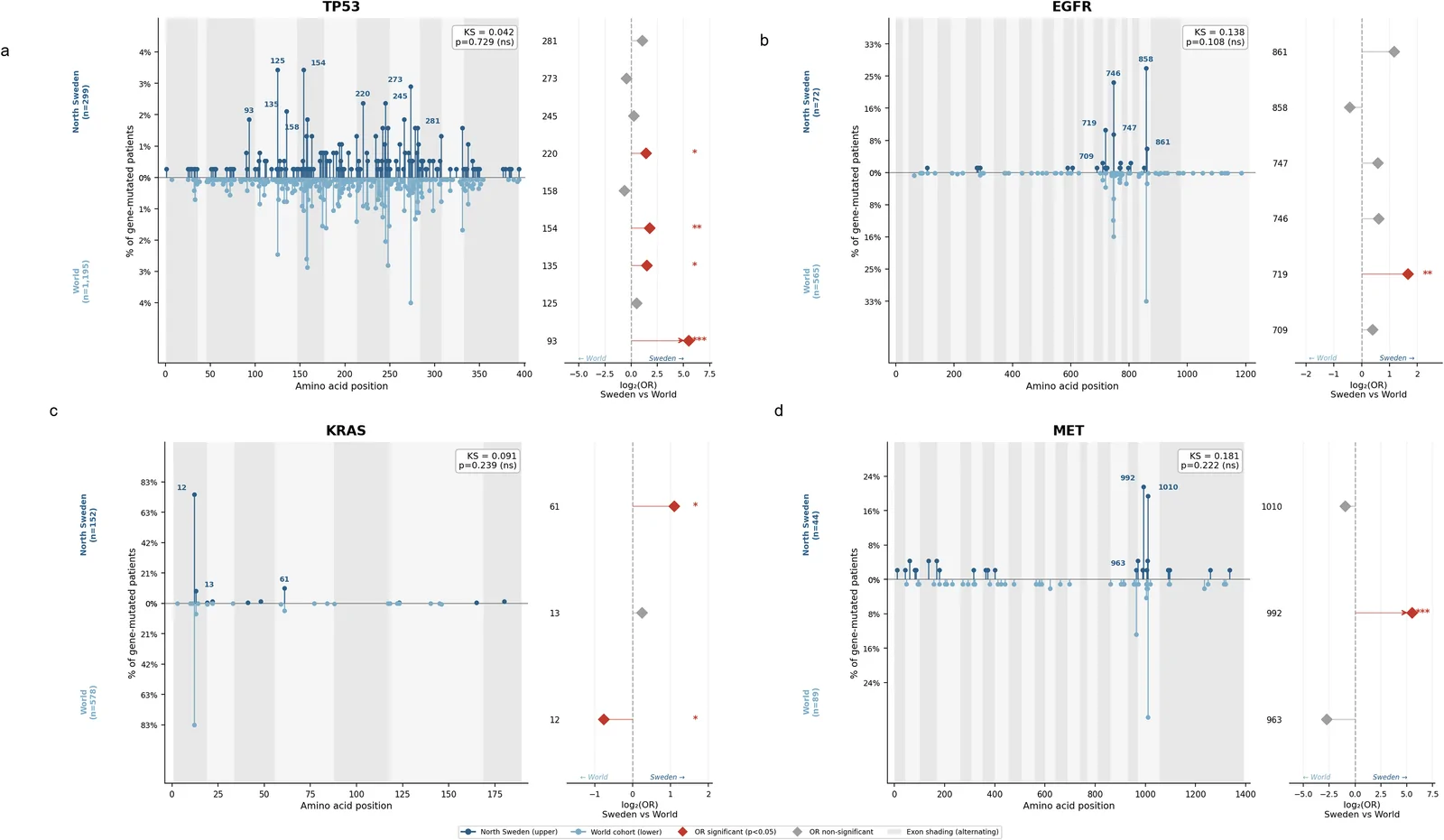
Positional architecture and cohort enrichment of recurrent *TP53, EGFR, KRAS*, and *MET* alterations. Panels **(A)** *TP53*, **(B)** *EGFR*, **(C)** *KRAS*, and **(D)** *MET*. Within each panel, the left plot shows mutation frequencies by amino-acid position among gene-mutated patients, with North Sweden plotted upward (dark blue) and the world cohort downward (light blue), and alternating shading marking exon boundaries; recurrently mutated positions are labelled. The right plot shows position-level enrichment as the log₂ odds ratio for North Sweden versus the world cohort, with red diamonds indicating positions significantly enriched or depleted in North Sweden (*p < 0.05, **p < 0.01, ***p < 0.001); the dashed line marks log₂(OR) = 0.

*EGFR* mutations were less common in Sweden than globally (13.64% vs 25.86% of patients), but among *EGFR*-mutated cases both cohorts retained the expected kinase-domain hotspot structure (**Figure 3B**), with prominent exon 19/L858R-centered patterns and only modest shifts in breakpoint/hotspot frequencies but the position-wise KS comparison was consistent with broadly similar overall profiles. Sweden showed a relative enrichment of G719X (12.50% vs 4.15% of *EGFR*-mutated cases; OR = 3.1), whereas baseline T790M (3.19%) and position 745 events were observed only in the global cohort.

*KRAS* prevalence was comparable (28.79% Sweden vs 25.82% global), with codon 12 dominant in both cohorts but slightly less so in Sweden (G12 in 76.32% of *KRAS*-mutated Swedish cases vs 84.43% globally; OR 0.59, p = 0.021) alongside a relative shift toward codon 61 (10.53% vs 5.19%; OR 2.15, p = 0.023) (**Figure 3C**). Within codon 12, subtype composition was closely matched between cohorts (G12C 46.55% vs 45.31% of G12; G12V 23.28% vs 21.63%; G12D 17.24% vs 16.12%), with Sweden showing slightly more G12V/G12S and the global cohort relatively more G12A. No statistically significant differences in G12 subtype distribution were observed between cohorts (all p > 0.05), indicating that the overall codon 12 spectrum and with it the potential patient pool for *KRAS* G12C-targeted therapies is broadly preserved in this northern European population (**Supplementary Figure 1C**).

*MET* alterations were more frequent in Sweden than globally (8.33% vs 3.97% of patients) and were concentrated in isolated substitutions and occasional splice-region/splice-site–type events in/near the exon 14 regulatory region (positions 963, 992 [T992I], and 1010 [D1010N]), without directly observed canonical exon 14 skipping events in this dataset (**Figure 3D**). Unlike *TP53*/*EGFR*/*KRAS*, *MET* (KS = 0.181, p = 0.222), largely driven by position 992, which accounted for 22.73% of *MET*-mutated Swedish cases and was not observed in the global series (OR 54.48, p = 7e-06); given the concentration at a single site and the predominance of missense calls, this enrichment is also compatible with a possible residual germline contribution Variant allele frequencies of T992I in Swedish cases were consistent with heterozygosity (median 0.46, range 0.26–0.69. The gnomAD population frequency of T992I is 2.0% in Finnish and 1.3% in non-Finnish European individuals compared with 0.004% in East Asian populations (**Supplementary Table 2A**). of 992, differences were smaller, with 1010 less frequent in Sweden (11.36% vs 33.71% OR 0.25, p = 0.0063) and 963 observed in the global cohort (13.48%) but not in Sweden (0%; OR 0.07, p = 0.0086).

### Prognostic markers

Survival analysis of the North Swedish cohort, revealed EGFR as the most significant positive prognostic marker, conferring a 24-month survival probability of 72.2% in EGFR-mutated patients vs 45.2% in non-mutated patients (+0.27, p < 0.001) (**Figure 4A**). TP53 also reached statistical significance in the North Sweden cohort, associated with a 24-month survival probability of 46.6% in TP53-mutated vs 51.7% in non-mutated patients (–0.05, p = 0.014), and RB1 was identified as another significant negative prognostic marker (30.0% vs 49.6% in non-mutated; –0.20, p = 0.027) (**Figure 4A**). In contrast, survival analysis identified a broader spectrum of significant genes in the global cohort. EGFR again showed a positive survival effect (88.3% vs 77.2%; +0.11, p < 0.001), while TP53 (75.6% vs 85.1% in non-mutated; –0.10, p < 0.001), APC (67.3% vs 81.0%; –0.14, p < 0.001), PIK3CA (72.3% vs 81.0%; –0.09, p < 0.001), FLT3 (69.1% vs 48.8%; –0.13, p = 0.003), CDKN2A (69.3% vs 81.1%; –0.12, p = 0.016), and STK11 (75.7% vs 81.0%; –0.05, p = 0.043) all emerged as significant negative prognostic markers (**Figure 4B**). Direct comparison of survival differences between cohorts (**Figure 4C**) highlighted that MET’s prognostic impact diverged, with a negative, non-significant effect in North Sweden (43.2% vs 49.4% in non-mutated patients; –0.06, p = 0.36) and a positive, significant effect in the world cohort (+0.08, p = 0.03), reflecting both its higher frequency and unique mutation spectrum in Sweden, particularly the enrichment of T992I substitutions. EGFR was associated with improved 24-month survival in both cohorts. In North Sweden, the absolute survival difference between EGFR-mutated and non-mutated patients was larger than in the world cohort (+0.27 vs +0.11), whereas the absolute 24-month survival of EGFR-mutated patients was lower in North Sweden than globally (72.2% vs 88.3%).

**Figure 4:**
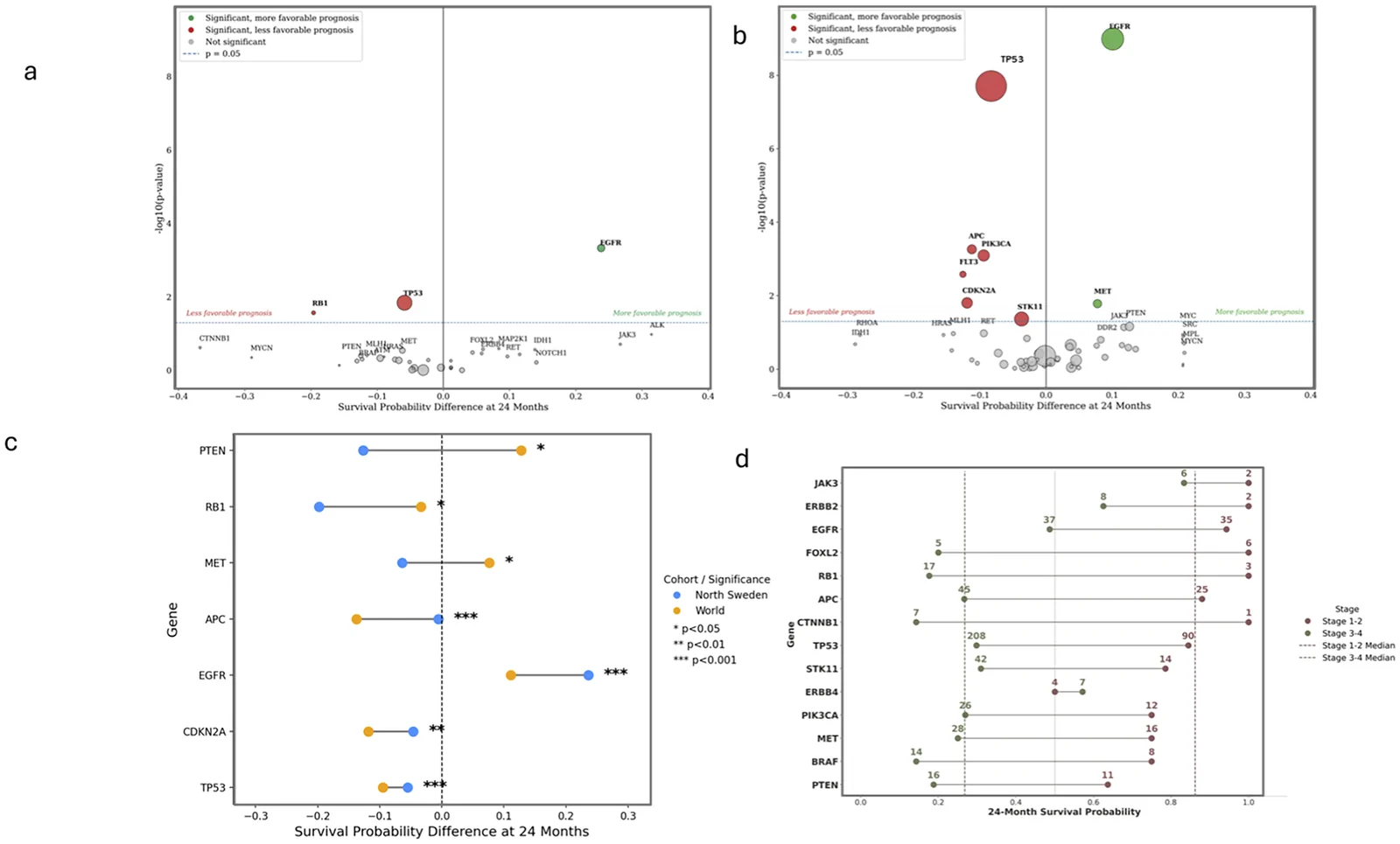
Gene-level associations between mutation status and 24-month overall survival in the North Swedish and world lung cancer cohorts. **(A, B)** show volcano plots for the North Swedish cohort (n=528) and the survival-linked world cohort (n=2,197), respectively. Each point represents one gene analyzed at the patient level. For each gene, patients with at least one mutation in that gene were compared with all other patients without that gene mutation. The x-axis shows the difference in Kaplan-Meier estimated 24-month overall survival probability (mutated minus non-mutated), and the y-axis shows -log10(p) from the two-sided log-rank test. Only genes mutated in at least 5 patients were included. Point size reflects the number of mutated patients, and genes with p < 0.05 are highlighted. **(C, D)** Forest plots illustrating 24-month survival probabilities for patients with mutations in selected genes. **(C)** compares the difference in 24-month survival probability for each gene between the North Swedish cohort (blue) and the rest of the world (yellow). Genes are shown on the y-axis, with the x-axis representing the difference in survival probability at 24 months. Dots represent the survival probability for each cohort, and horizontal lines connect the two cohorts for each gene. Significance stars (*, **, ***) indicate statistical significance of the difference in survival probability (p < 0.05, p < 0.01, p < 0.001, respectively). **(D)** shows the 24-month survival probability for the Swedish cohort, stratified by stage group (Stage 1-2: blue; Stage 3-4: orange). The number above each dot indicates the number of unique patients in each gene/stage, and horizontal lines connect the two stage groups for each gene.

To distinguish whether poor survival in certain gene groups was due to enrichment of late-stage patients or reflected stage-independent effects, we compared 24-month survival probabilities for each gene mutation within early (stage 1–2) and advanced (stage 3–4) disease, using the pooled patient-level median for each stage as a reference (**Figure 4D**). This analysis revealed marked heterogeneity in which EGFR-mutated patients (n=72) had a large early stage patient distribution (49% stage 1–2, 51% stage 3–4), while RB1 (n=20) and CTNNB1 (n=8) showed the lowest survival (0.30 and 0.25), predominantly in advanced stage (RB1: 85%, CTNNB1: 88% stage 3–4). JAK3 mutations (n=8) conferred the highest survival (0.88) despite 75% being late stage, and ERBB2 (n=10) and ERBB4 (n=11) also showed high survival (0.70 and 0.64) with most patients in stage 3–4 (**Figure 4D**; **Supplementary Figure 1D**). Notably, EGFR, ERBB2, and JAK3 groups all exhibited a significant drop in 24-month survival probability among stage 3–4 patients compared to stage 1–2 (EGFR: 0.94 vs. 0.49, p < 0.01). In contrast, PTEN (0.41, 59% stage 3–4) and BRAF (0.45, 64% stage 3–4) were associated with poor survival not fully explained by stage, suggesting possible stage-independent adverse effects.

### Comparative enrichment analysis

Across the Swedish cohort, subgroup-skewed mutation patterns were most coherent for *EGFR* and for alterations linked to smoking exposure. *EGFR* showed the clearest and most consistent enrichment pattern, with higher prevalence in females than males (log2FC=1.40, p = 6.46×10^−^5) and lower prevalence in smokers than never smokers (Smoker vs Never Smoker log2FC=−1.75, p=4.11×10^−^7) (**Figure 5A**). *EGFR* was also more frequent in patients below the median age (log2FC=0.83, p = 0.0107) and in early-stage disease (Late vs Early log2FC=−0.82, p = 0.0111), supporting a clinically coherent *EGFR*-associated subtype rather than a marker of generalized progression. In contrast, smoking-associated differences were driven by canonical tobacco-linked genes, with *KRAS* and *STK11* enriched in smokers (log2FC=0.93, p = 0.00608 and log2FC =1.89, p = 0.014), alongside an increase in *TP53* in smokers (log2FC=0.53, p = 0.00385) (**Figure 5A**). Stage-related shifts were more modest overall, but *TP53* was more frequent in late-stage tumours than early-stage tumours (log2FC=0.31, p = 0.0125). Age stratification highlighted a PI3K-pathway skew, with *PIK3CA* enriched in older patients (Below vs Above median log2FC=−1.44, p = 0.00369). Several additional large-effect directional patterns were apparent in the heatmap but did not meet the significance threshold such as a strong smoker-skew for *FBXW7* (Smoker vs Never Smoker log2FC=2.73), consistent with limited power in low-count subgroups.

**Figure 5:**
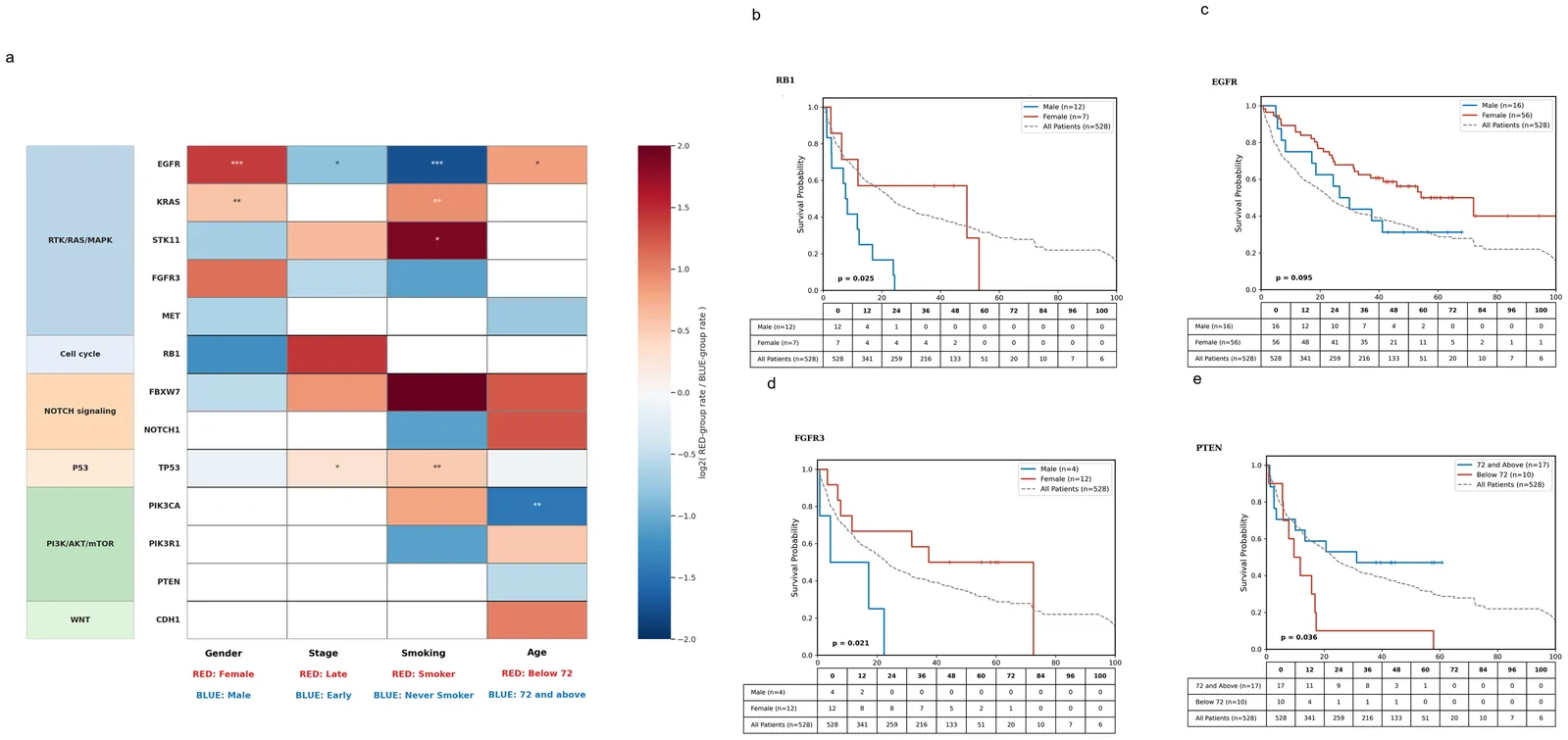
Mutation enrichment across clinical subgroups and exploratory subgroup survival analyses in the North Swedish cohort. **(A)** Heatmap of log_2_ fold-change in recurrent mutation frequencies across four clinical comparisons: sex (female vs male), stage (late III–IV vs early I–II), smoking history (ever-smoker vs never-smoker), and age (below vs above cohort median of 72 years). Red shading indicates enrichment in female, late-stage, ever-smoker, or younger patients; blue shading indicates enrichment in male, early-stage, never-smoker, or older patients. Only genes with |log_2_FC| > 0.5 and/or p < 0.05 in at least one comparison are shown. **(B–E)** Kaplan–Meier curves showing overall survival within selected gene-mutated subgroups: **(B)** *RB1* by sex (12 males vs 7 females); **(C)** *FGFR3* by sex (4 males vs 12 females); **(D)** *EGFR* by sex (16 males vs 56 females); and **(E)** *PTEN* by age (<72 years vs ≥72 years). P values are two-sided log-rank tests. Numbers at risk are shown beneath each panel at defined time intervals.

Kaplan–Meier survival analysis was performed for *FGFR3*, *EGFR*, and *RB1* mutations, stratified by sex, in both the North Swedish cohort and the world reference dataset. For *RB1*, the mutated patients trended towards more common in males in the North Swedish cohort (12 males vs 7 females, additionally *RB1*-mutant males had significantly worse survival than females (p = 0.025; **Figure 5B**), this difference in survival between sex was also observed in the world cohort (58 males vs 59 females, p = 0.008; **Supplementary Figure 2A**). For *EGFR*, patients were significantly enriched in females in North Sweden (56 females vs 16 males, p = 6.46×10^−^5), but survival trended toward worse outcomes for males and although this difference was not statistically significant in North Sweden (p = 0.095; **Figure 5C**), it reached significance in the larger world cohort (p = 0.038) (**Supplementary Figure 2B**). A similar pattern was observed for *FGFR3*, where the mutation was more frequent in females (12 females vs 4 males), yet males had worse survival (p = 0.021; **Figure 5D**; **Supplementary Figure 2C**).

Overall survival worsened with increasing age, with patients aged 72 and above showing significantly poorer survival than those below 72 (log-rank p = 0.00152; **Supplementary Figure 3**) In this context, *PTEN* showed an unusual age-related pattern. Among younger patients, *PTEN*-mutant cases had poorer survival in the North Swedish cohort (p = 0.036; **Figure 5E**), although the trend was not mirrored in the world cohort, (**Supplementary Figure 2D**).

## Discussion

The promise of precision oncology rests partly on the assumption that genomic profiles translate uniformly into clinical outcomes, yet mounting evidence suggests that regional, demographic, and environmental factors profoundly shape which mutations arise and how they behave. The management of lung cancer is at present mainly focused on detecting specific, actionable gene mutations, often overlooking these broader contextual influences on tumour heterogeneity. With advances in genetic testing, routine profiling can be used not only to detect actionable alterations, but also to understand how environmental exposures, population-level differences, and co-mutation patterns shape tumour biology and clinical outcome (19).

In our cohort from North Sweden, a relatively homogeneous population, routine clinical sequencing of 67 key genes in 528 patients identified 1,415 somatic mutations. This revealed substantial genetic diversity, even within our region. By further stratifying patients by demographic and lifestyle attributes such as age, sex, and smoking status showed that the clinical meaning of recurrent mutations depends strongly on context.

We observed a wide range in the number of mutations per patient, from none to 44, underscoring the heterogeneity of lung tumours in our cohort. Although the number of mutations did not clearly correlate with survival, tumours with higher mutation counts may be more resistant to therapy, while those with fewer mutations could respond better to targeted treatments, though hidden subclones may still drive disease progression (20). Smaller subgroups were driven by single dominant oncogenic events (e.g., *BRAF*, *ALK*, *FGFR2*/3) with relatively few co-alterations, while the broader landscape showed stable common drivers across stages and only low-frequency late-stage enrichment of rarer genes (e.g., *EZH2*, *VHL*, *RHOA*), consistent with progression shaped more by genomic instability than simple time-dependent accumulation (21).

Across cohorts, the dominant driver genes showed broadly conserved hotspot architectures, but clinically meaningful differences emerged in which specific variants were enriched. In *EGFR*, North Sweden had a lower overall prevalence (implying fewer candidates for *EGFR*-directed therapy), yet *EGFR* mutations still clustered in canonical activating regions (22). The relative enrichment of G719X mutations which confer sensitivity to second-generation EGFR inhibitors such as afatinib (23) highlights the need for routine workflows to capture less common sensitizing variants rather than focusing narrowly on exon 19 deletions and L858R (23). In the survival analyses, *EGFR* remained a positive prognostic marker in both cohorts however, in North Sweden its apparent benefit was clearly attenuated in advanced disease, with a substantial reduction in 24-month survival for stage 3–4 versus stage 1–2 patients (0.94 vs 0.49). As EGFR-mutant patients in routine practice would be expected to receive EGFR-directed TKI therapy, the observed survival advantage likely reflects both the stage distribution of this subgroup and the efficacy of targeted treatment rather than an intrinsic tumour biology effect. Baseline *EGFR* T790M was detected only in the global cohort and not in North Sweden, a difference that may be clinically relevant because T790M can reduce sensitivity to first-/second-generation *EGFR* TKIs, but should be interpreted cautiously in the absence of treatment data in either cohorts (24).

*TP53* mutations clustered in the DNA-binding domain in both cohorts, and the Swedish enrichments at R154 and Y220 reflect localised hotspot shifts rather than a fundamentally different mutational biology (25). *TP53* retained a reproducible negative prognostic signal across datasets. *KRAS* codon 12 dominance was broadly preserved, with G12C the most common subtype in both cohorts, and its frequency in our region is consistent with national Swedish registry data, supporting the use of G12C inhibitors such as sotorasib and adagrasib in this population (26, 27). *MET* was the clearest regional outlier with higher frequency in North Sweden cohort (8.3% vs 4.0%) and dominated by T992I substitutions near the exon 14 regulatory region rather than canonical exon 14 skipping events (28). This tight clustering at a single site represents the most striking site-specific finding in this cohort and warrants formal investigation to distinguish a shared somatic hotspot from a recurrent germline variant escaping filtering. *MET*’s divergent prognostic behaviour between cohorts further suggests that variant spectrum, not alteration status, drives clinical outcomes (29, 30). *APC* was also markedly enriched in Sweden (13.3% vs 4.6%, p < 0.001), a canonical Wnt/β-catenin tumour suppressor whose somatic inactivation in lung cancer is less well characterised than in colorectal cancer (31). Its negative prognostic signal globally but not locally may reflect different co-mutation contexts. *ATM* enrichment (10.8% vs 6.6%) is clinically relevant given evidence that ATM-mutant NSCLC shows distinct immunophenotypic features and may have therapeutic relevance for DNA damage response-targeted strategies (32). Neither enrichment is readily explained by known demographic factors, and both merit prospective validation.

Poor survival in several gene-mutant groups is primarily explained by their enrichment in advanced-stage disease rather than an independent molecular effect, while other gene groups carry adverse prognosis through mechanisms less easily explained by stage distribution alone. This distinction has direct clinical implications where stage-independent markers such as PTEN and BRAF may warrant prospective evaluation as independent prognostic biomarkers and treatment decisions, whereas stage-enriched markers (e.g., RB1, CTNNB1) primarily reflect disease severity rather than a targetable molecular event. This pattern converges with the world cohort findings where PIK3CA, STK11, and CDKN2A (genes involved in PI3K signalling, metabolic stress tolerance, and cell-cycle checkpoint control) emerge as additional adverse markers, together suggesting that disruption of these integrated cell survival pathways confers prognosis beyond what stage alone can explain (33). *RB1* and *CTNNB1* concentrate heavily in advanced-stage patients (85% and 88% stage 3–4, respectively), suggesting their apparent prognostic impact may at least partly reflect disease burden at presentation rather than intrinsic tumour aggressiveness per se (33, 34). Conversely, several small subgroups displayed unexpectedly favourable 24–month survival despite predominantly late-stage disease. JAK3 showed the most favourable 24-month survival despite predominantly advanced-stage disease, although the small patient number (n=8) precludes firm conclusions. *ERBB2* (0.70) and *ERBB4* (0.64) showed similarly encouraging survival profiles, possibly reflecting off-label use of HER2-directed therapies in this small patient group. Within each of these groups, stage 3–4 still carried substantially worse survival than stage 1–2, underscoring that stage remains the dominant prognostic axis across genotypes. In contrast, *PTEN* (0.41; 59% stage 3–4) and *BRAF* (0.45; 64% stage 3–4) showed poor survival not obviously proportional to their stage distributions, pointing to possible stage-independent adverse effect.

Sex and smoking status define biologically coherent mutation subgroups, with *EGFR* clustering in never-smoker, younger, and female patients, and *KRAS*, *STK11*, and *TP53* enriched in smoking-associated disease, consistent with established NSCLC subtype structure (35). Notably, *TP53* also showed additional enrichment in late-stage disease, consistent with its association with more advanced or treatment-resistant tumour states. The strong smoker-associated enrichment of *STK11* in our cohort is of particular clinical relevance, since *STK11*/LKB1 loss is one of the major drivers of primary immunotherapy resistance in NSCLC and is associated with an immunologically cold tumour microenvironment (36). Given that the majority of our cohort comprises ever-smokers, *STK11* co-mutations may contribute to variable immunotherapy responses in this population and should be considered alongside PD-L1 status in treatment planning (37). *EGFR* was enriched in females and never-smokers, supporting a distinct *EGFR*-driven subtype. However, survival among EGFR-mutant cases was poorer in males, reaching significance in the larger world cohort, suggesting that the prognostic effect of EGFR mutation is not uniform across sex. *FGFR3* provided a second example where prevalence and prognosis diverged where although *FGFR3* mutations were more common in women, men with these mutations had significantly worse survival in Sweden. This may reflect androgen-related modulation of FGFR3-driven tumour biology (38, 39).The most consistent sex–genotype interaction is seen in *RB1*, where the male skewing of mutation prevalence and worse *RB1*-mutant survival in males, replicated across both the North Swedish and world cohorts, together point to sex-linked biology amplifying the clinical consequences of cell-cycle checkpoint loss. This sex-specific survival pattern may be influenced by androgen-related signalling and differences in immune surveillance (40). Beyond its role as an independent prognostic variable, older age is associated with a distinct shift in the mutational landscape, most notably toward PI3K pathway alterations, while conversely certain mutations carry worse survival specifically in younger patients. The enrichment of *PIK3CA* mutations in older patients is consistent with previous reports showing that patients with *PIK3CA*-mutant NSCLC tend to be older than those without *PIK3CA* mutations (41). This suggests that PI3K pathway alterations may accumulate over time. *PTEN* showed a different pattern. Although *PTEN* mutations were more frequent in older patients overall, *PTEN*-mutant cases had markedly worse survival among younger patients in North Sweden (p = 0.036). This may reflect particularly aggressive tumour behaviour driven by unchecked PI3K/AKT/mTOR activation in biologically younger and more proliferatively active tumours, a context in which *PTEN* loss removes a critical brake on cell cycle progression (42, 43).

This real-world study is based on a regional consecutive cohort from one region served by a single university hospital which is a strength limiting selection bias in the north Swedish cohort. However, the study design also have several limitations which warrant acknowledgement such as the absence of treatment data means prognostic associations should be interpreted as observational, MET T992I clustering at a single site may reflect residual germline contribution which should be validated, age data were missing for 41.6% of the world cohort and several gene subgroup analyses involve small numbers and should be considered hypothesis-generating pending independent replication. Some of these findings include small number subgroups and should be considered hypothesis-generating pending independent validation.

Together, these findings highlight how the lung cancer mutational landscape and its clinical consequences emerge from a layered interplay between molecular drivers, environmental exposure (smoking), and clinical context (stage, age, and sex), even within a relatively homogeneous regional population. Subgroup enrichment clarifies who carries key alterations, but prognosis reflects the combined effects of genotype, host biology, and care pathways, with enrichment and outcome sometimes aligning (RB1) and sometimes diverging (EGFR, FGFR3). These insights underscore the value of integrating clinical variables such as age, sex, and stage alongside genomic data to refine risk stratification and guide more personalised management strategies.

## Data Availability

The North Sweden genomic and clinical data analysed in this study are derived from routine regional diagnostic records and registry linkage and are not publicly available due to Swedish data-protection legislation and the terms of the ethical approval (patient confidentiality). Requests to access the datasets should be directed to the corresponding author.
