# Correction: Real world genomic profiling in non-small cell lung cancer reveals regional variations with clinical implications

**DOI:** 10.3389/fonc.2026.2013767

**Published:** 2026-10-09

**Authors:** Frontiers Production Office

**Affiliations:** Frontiers Media SA, Lausanne, Switzerland

**Keywords:** clinical genomics, lung cancer, mutational landscape, North Sweden, precision oncology, somatic mutation, targeted profiling

There was an error in **Figure 4** as published. The image displayed as **Figure 4** is **Supplementary Figure 2**, which was wrongly published in place of **Figure 4**. The caption of the published **Figure 4** was correct and does not need to be changed. The corrected **Figure 4** appears below.

**Figure 4:**
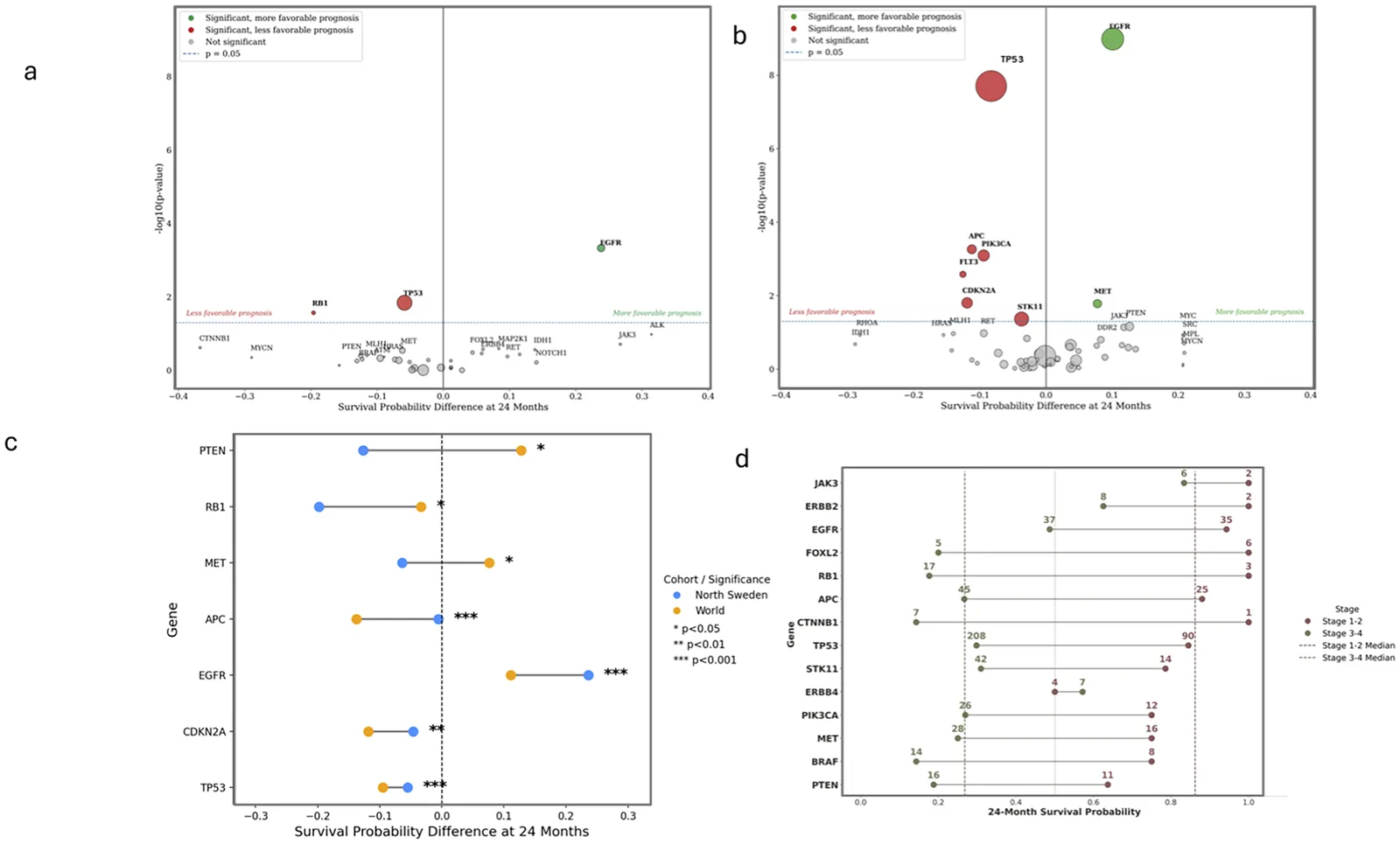
Gene-level associations between mutation status and 24-month overall survival in the North Swedish and world lung cancer cohorts. **(A, B)** show volcano plots for the North Swedish cohort (n=528) and the survival-linked world cohort (n=2,197), respectively. Each point represents one gene analyzed at the patient level. For each gene, patients with at least one mutation in that gene were compared with all other patients without that gene mutation. The x-axis shows the difference in Kaplan-Meier estimated 24-month overall survival probability (mutated minus non-mutated), and the y-axis shows -log10(p) from the two-sided log-rank test. Only genes mutated in at least 5 patients were included. Point size reflects the number of mutated patients, and genes with p < 0.05 are highlighted. **(C, D)** Forest plots illustrating 24-month survival probabilities for patients with mutations in selected genes. **(C)** compares the difference in 24-month survival probability for each gene between the North Swedish cohort (blue) and the rest of the world (yellow). Genes are shown on the y-axis, with the x-axis representing the difference in survival probability at 24 months. Dots represent the survival probability for each cohort, and horizontal lines connect the two cohorts for each gene. Significance stars (*, **, ***) indicate statistical significance of the difference in survival probability (p < 0.05, p < 0.01, p < 0.001, respectively). **(D)** shows the 24-month survival probability for the Swedish cohort, stratified by stage group (Stage 1-2: blue; Stage 3-4: orange). The number above each dot indicates the number of unique patients in each gene/stage, and horizontal lines connect the two stage groups for each gene.

**Supplementary Figure 3**, which is cited in the **Results** section, was erroneously omitted from the **Supplementary Material** as published. The **Supplementary Material** has been updated to include **Supplementary Figure 3**.

The original version of this article has been updated.

