# Supplementary material for: Real world genomic profiling in non-small cell lung cancer reveals regional variations with clinical implications
Source: Front Oncol. 2026 Sep 22;16:1882423. doi: 10.3389/fonc.2026.1882423 (PMC13638359; doi:10.3389/fonc.2026.1882423)
Supplement: Supplementary file 1 [file DataSheet1.pdf]

Supplementary Material

Supplementary Figure 1 | (A) Violin plots showing somatic mutation burden (mutations per patient) across clinical stage groups (stages I-IV) for the North Swedish cohort (left; Kruskal-Wallis $p = 0.817$) and the world reference cohort (right; Kruskal-Wallis $p = 0.396$). Mutation burden did not differ significantly across stages in either cohort. (B) Composition of the world reference dataset (n = 2,239); segments indicate the proportion of patients from each of the five contributing studies. (C) Distribution of KRAS codon 12 variant subtypes among KRAS-mutated patients in the North Swedish cohort (n = 116) and the world cohort (n = 488). Bars show the proportion harbouring each G12 substitution. (D) Stacked bars show the proportion of gene-mutated patients with early (Stage I-II) versus late (Stage III-IV) disease for each gene in Figure 4D, ordered by 24-month overall survival. The dashed line marks the cohort background. Fisher's exact test; * $p < 0.05$, ** $p < 0.01$. (E) Somatic mutational landscape of the world reference cohort (n = 2,239). Each column represents one patient and each row one gene, ordered by decreasing mutation frequency. Mutation categories are colour-coded as in Figure 2A. Clinical annotation tracks for disease stage, sex, and smoking history are shown beneath the matrix.

Supplementary Figure 2 | Exploratory subgroup survival analyses in the world cohort, mirroring Figure 5B-E. Kaplan-Meier curves show overall survival within selected gene-mutated subgroups: (A) RB1 by sex (male n = 58, female n = 59; log-rank $p = 0.008$); (B) EGFR by sex (male n = 161, female n = 408; $p = 0.038$); (C) FGFR3 by sex (male n = 11, female n = 10; $p = 0.952$); and (D) PTEN by age group (aged >=72 years n = 34, aged <72 years n = 39; $p = 0.063$). P values are two-sided unadjusted log-rank tests. Numbers at risk are shown beneath each panel.

Supplementary Figure 3 | Overall survival stratified by age group in the North Swedish cohort, using the cohort median of 72 years as the cut-point. Kaplan-Meier curves are shown for patients aged <72 years (n = 246) and >=72 years (n = 282; log-rank $p = 0.00152$). Numbers at risk are displayed beneath the panel.

Supplementary Table 1A | Baseline characteristics of the global lung cancer cohort (N = 2,239)

Shown are distributions of age, disease stage, histological subtype, and survival, stratified by sex.

Characteristic	Total (N=2239)	Male (n=1028)	Female (n=1211)
**Demographics**			
Age, mean (median), years	65.69 (67)	66.04 (67)	65.26 (66)
Age below mean (65.69)	592 (26.4%)	310 (30.2%)	282 (23.3%)
Age above mean (65.69)	715 (31.9%)	398 (38.7%)	317 (26.2%)
Age unspecified	932 (41.6%)	320 (31.1%)	612 (50.5%)
**Disease Stage**			
Stage I	600 (26.8%)	313 (30.4%)	287 (23.7%)
Stage II	313 (14.0%)	206 (20.0%)	107 (8.8%)
Stage III	243 (10.9%)	145 (14.1%)	98 (8.1%)
Stage IV	50 (2.2%)	32 (3.1%)	18 (1.5%)
Stage unspecified	1033 (46.1%)	332 (32.3%)	701 (57.9%)
**Histological Subtype**			
Adenocarcinoma	1783 (79.6%)	688 (66.9%)	1095 (90.4%)
Squamous cell carcinoma	456 (20.4%)	340 (33.1%)	116 (9.6%)
**Vital Status**			
Alive	1628 (72.7%)	711 (69.2%)	917 (75.7%)
Deceased	610 (27.2%)	316 (30.7%)	294 (24.3%)
Unspecified	1 (0.0%)	1 (0.1%)	0 (0.0%)
Median overall survival, months	24.8	20.6	27.7

Supplementary Table 1B | Mapping of raw consequence annotations to broad mutation classes

Unique database consequence strings were grouped into broad descriptive mutation classes.

Mutation Category	Included Consequence String(s)	Interpretation
Missense Variant	missense_variant; missense_variant&splice_region_variant; protein_altering_variant; stop_lost	Protein-altering substitutions not classified as truncating or inframe events.
Loss of Function	frameshift_variant; stop_gained; splice_acceptor_variant; splice_donor_variant and equivalent truncating/canonical splice strings	Truncating and canonical splice-disrupting alterations.
Inframe Variant	inframe_deletion; inframe_insertion	In-frame insertions or deletions.
Regulatory Variant	upstream_gene_variant; downstream_gene_variant; splice_region_variant outside canonical donor/acceptor loss	Non-coding or splice-region events outside canonical splice loss.
Other/Unknown	Other uncategorized source entries	Unclassified source entries retained as other/unknown.
