# Supplementary material for: Real world genomic profiling in non-small cell lung cancer reveals regional variations with clinical implications
Source: Front Oncol. 2026 Sep 22;16:1882423. doi: 10.3389/fonc.2026.1882423 (PMC13638359; doi:10.3389/fonc.2026.1882423)
Supplement: Supplementary file 2 [file SupplementaryFile1.pdf]

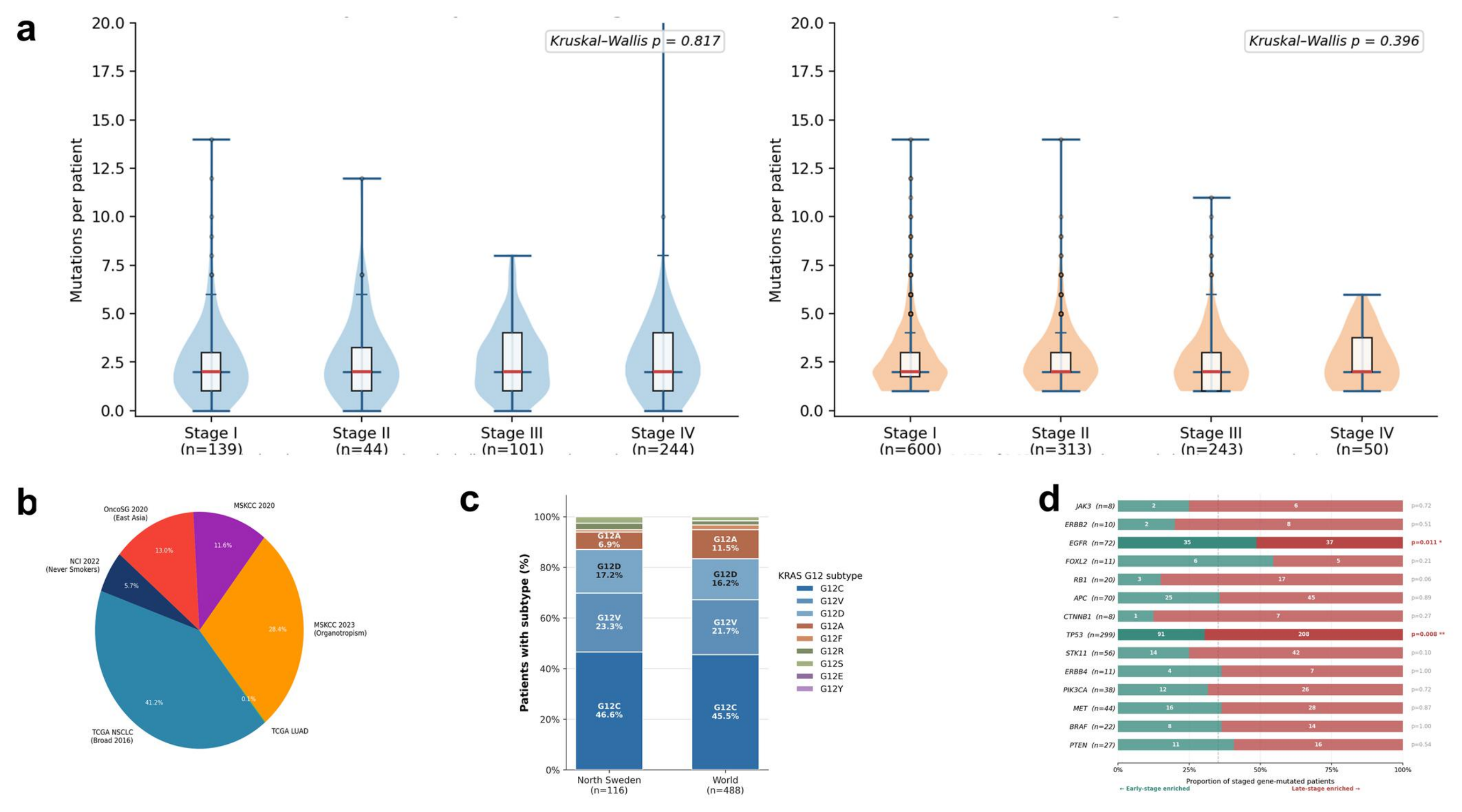

a
Kruskal–Wallis p = 0.817
Mutations per patient
20.0
17.5
15.0
12.5
10.0
7.5
5.0
2.5
0.0
Stage I (n=139)
Stage II (n=44)
Stage III (n=101)
Stage IV (n=244)
Kruskal–Wallis p = 0.396
Mutations per patient
Stage I (n=600)
Stage II (n=313)
Stage III (n=243)
Stage IV (n=50)
b
MSKCC 2020
OncoSG 2020 (East Asia)
NCI 2022 (Never Smokers)
MSKCC 2023 (Organotropism)
TCGA NSCLC (Broad 2016)
TCGA LUAD
13.0%
11.6%
5.7%
28.4%
41.2%
0.1%
c
Patients with subtype (%)
100%
80%
60%
40%
20%
0%
G12A 6.9%
G12D 17.2%
G12V 23.3%
G12C 46.6%
G12A 11.5%
G12D 16.2%
G12V 21.7%
G12C 45.5%
North Sweden (n=116)
World (n=488)
KRAS G12 subtype
G12C
G12V
G12D
G12A
G12F
G12R
G12S
G12E
G12Y
d
JAK3 (n=8) 2 6 p=0.72
ERBB2 (n=10) 2 8 p=0.51
EGFR (n=72) 35 37 p=0.011 *
FOXL2 (n=11) 6 5 p=0.21
RB1 (n=20) 3 17 p=0.06
APC (n=70) 25 45 p=0.89
CTNNB1 (n=8) 1 7 p=0.27
TP53 (n=299) 91 208 p=0.008 **
STK11 (n=56) 14 42 p=0.10
ERBB4 (n=11) 4 7 p=1.00
PIK3CA (n=38) 12 26 p=0.72
MET (n=44) 16 28 p=0.87
BRAF (n=22) 8 14 p=1.00
PTEN (n=27) 11 16 p=0.54
0%
25%
50%
75%
100%
Proportion of staged gene-mutated patients
← Early-stage enriched
Late-stage enriched →
