# Supplementary material for: Real world genomic profiling in non-small cell lung cancer reveals regional variations with clinical implications
Source: Front Oncol. 2026 Sep 22;16:1882423. doi: 10.3389/fonc.2026.1882423 (PMC13638359; doi:10.3389/fonc.2026.1882423)
Supplement: Supplementary file 3 [file SupplementaryFile2.pdf]

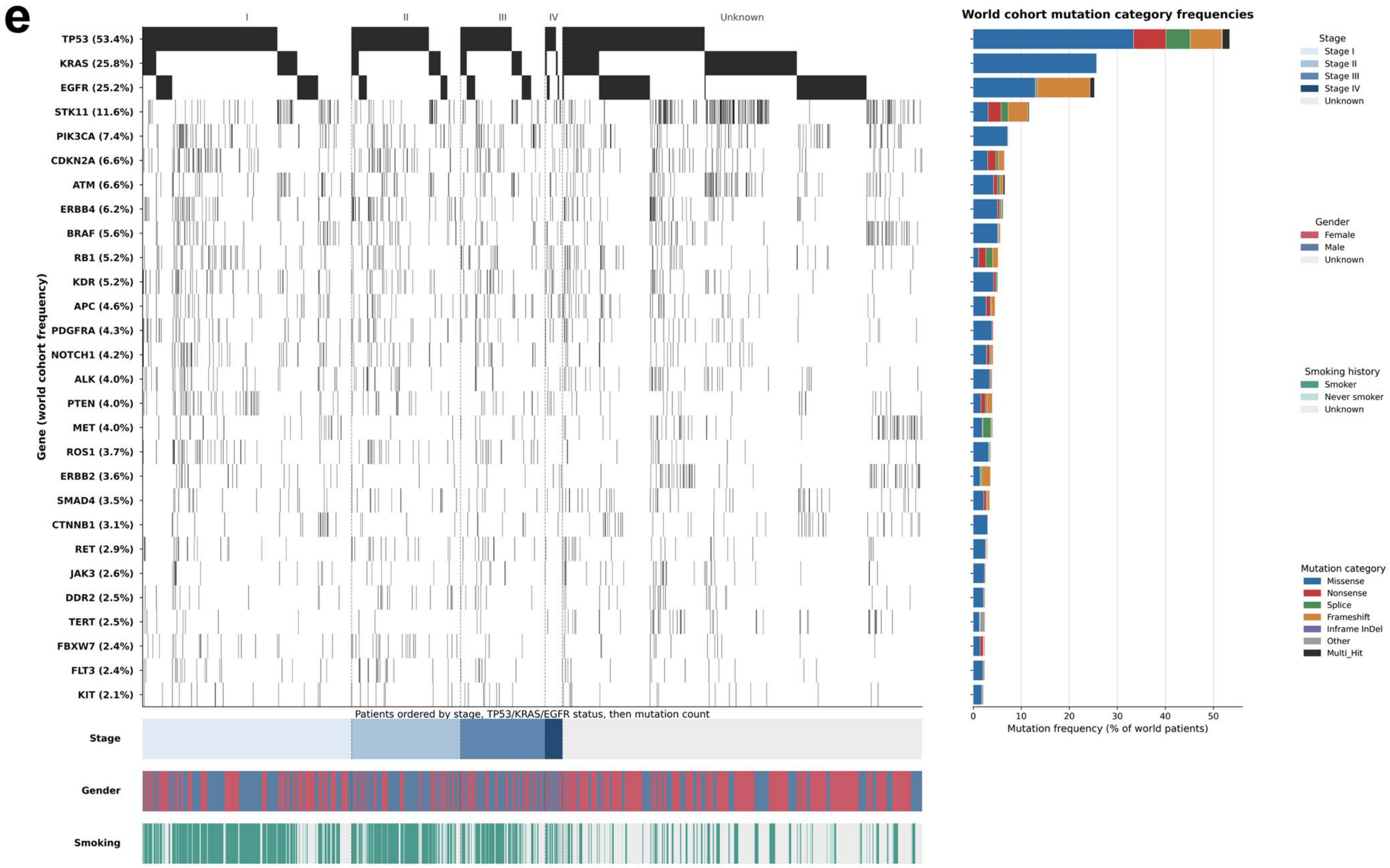
e
I
II
III
IV
Unknown
TP53 (53.4%)
KRAS (25.8%)
EGFR (25.2%)
STK11 (11.6%)
PIK3CA (7.4%)
CDKN2A (6.6%)
ATM (6.6%)
ERBB4 (6.2%)
BRAF (5.6%)
RB1 (5.2%)
KDR (5.2%)
APC (4.6%)
PDGFRA (4.3%)
NOTCH1 (4.2%)
ALK (4.0%)
PTEN (4.0%)
MET (4.0%)
ROS1 (3.7%)
ERBB2 (3.6%)
SMAD4 (3.5%)
CTNNB1 (3.1%)
RET (2.9%)
JAK3 (2.6%)
DDR2 (2.5%)
TERT (2.5%)
FBXW7 (2.4%)
FLT3 (2.4%)
KIT (2.1%)
Gene (world cohort frequency)
Patients ordered by stage, TP53/KRAS/EGFR status, then mutation count
Stage
Gender
Smoking
World cohort mutation category frequencies
0
10
20
30
40
50
Mutation frequency (% of world patients)
Stage
Stage I
Stage II
Stage III
Stage IV
Unknown
Gender
Female
Male
Unknown
Smoking history
Smoker
Never smoker
Unknown
Mutation category
Missense
Nonsense
Splice
Frameshift
Inframe InDel
Other
Multi_Hit
